# The Book World of Medicine and Science

**Published:** 1899-05-27

**Authors:** 


### The Book World of Medicine and Science.

A System of Medicine by Many Writers. Edited by Thomas Clifford Allbutt, M.A., M.D., LL.D., F.R.C.P., F.R.S., Regius Professor of Physic in the University of Cambridge. (London: Macmillan and Co. 1889. Vol. VI. Price 25s.)

Second Notice.

One side of the nervous system is so intimately related to the muscular system, by aid of which alone its activities find expression, that in regard to some diseases it is a little difficult to draw the line between them, or to say with definiteness on which side of that line the original lesion lies. Hence, perhaps, a little confusion in the arrangement of this volume, which, however, is of no serious importance. Passing over diseases of the muscles, we find that Dr. Bevan Lewis contributes an article on the general pathology of the nervous system. He commences by a description of the recent discoveries in the physiology of the nervous system, and gives certain definitions in regard to the relations to the various parts of the neuron, a proper understanding of which is necessary to the reader. It is but a short exposition, nevertheless it puts the matter very neatly, and renders clear much which without it might seem obscure to those not conversant with the drift of recent investigations.

In regard to the pathological bearings of the details described, he points out that the morphological unity of the neuron and the doctrine of contiguity rather than continuity of related neurons, favour a strict limitation of degenerative changes, arising in a nerve chain, to the individual neuron affected. "Thus in motor neurons, where the trophic nerve (*i.e.*, the cell), or the axon emerging from it, is affected to a minor degree, we get a primary parenchymatous degeneration. Should the lesion, however, be destructive of the cell, or the axon be severed, the more active or Wallerian degeneration ensues—in both instances confined to the neuron affected. In like manner focal lesions of the motor cortex, medulla, or cord lead to a secondary parenchymatous degeneration, which exhibits the same tendency to systemic limitation. In the next place, the cell being the trophic centre of the emergent axon, the distal parts of the latter or terminal arborisations will suffer first when such trophic influence is arrested. Similarly tabes dorsalis affords us an illustration of the degeneration of a sensory neuron formed by the ganglion cell of the posterior root, the peripheral sensory root, and the centripetal fibre in the posterior column of the cord."

Dr. Sherrington gives a very interesting and explanatory article on tremor, "Tendon phenomenon," and spasm, dealing with the physiological side, while Dr. Seymour Sharkey deals with the clinical and pathological sides of the same questions. The latter article deserves careful reading, showing as it does the very considerable number of conditions in which the activity of the knee-jerks may be modified. Dr. Barlow's article on Raynaud's Disease is one of great interest and importance. Not only is his description of the clinical phenomena very complete, but his remarks on the nosological affinities of the condition suggest how widespread may be the effects of disorders of the vaso-motor nerves. He groups under the heading of Raynaud's Disease three clinical groups of cases, namely, local syncope, local asphyxia, and symmetrical gangrene, which have the following features in common —(*a*) a temporary but recurrent morbid alteration in the blood supply and in the consequent nutrition of the extremities, and in some instances also of certain internal structures; (*b*) circulatory and nutritive changes, generally affecting similar parts on the two sides, though the changes are not necessarily equal in extent on both, and, exceptionally, the final manifestations may even be unilateral; (*c*) a spasmodic and recurrent contraction of the arterioles —as is generally maintained—supplying the parts concerned and causing the morbid changes. In the local treatment of these conditions galvanism appears to be of considerable service. Dr. Barlow's recommendation in regard to the application of the current is as follows: Immerse the extremity of the limb which is the subject of the local asphyxia in a large basin containing salt and tepid water; one pole of a constant battery is placed in contact with the upper part of the limb above the level of the water, and the other pole in the basin, thus converting the salt and water into an electrode. As many elements as the patient can comfortably bear should be employed; and the current should be made and broken at frequent intervals, so as to get repeated moderate contractions of the limb. The patient should also be instructed to make voluntary movements of the digits whilst the galvanism is applied. Erythromelalgia, on which Dr. Barlow also writes, is a condition which is of considerable interest, not only for its own sake, but because of the analogy of some of its symptoms with what occurs in the course of many inflammatory conditions. The disease is defined as "a chronic disease in which a part or parts of the body—usually one or more extremities—suffer with pain, flushing, and local fever, made far worse if the parts hang down." The relations of this condition and its possible causes are fully discussed.

A very full account is given by Dr. Gibson and Dr. Fleming of the diseases of the spinal nerves; the effects of neuritis, tumours, pressure, and wounds being first dealt with, after which the individual affections of the different nerves are described.

The very large subject of symmetrical peripheral neuritis is dealt with by Dr. Judson Bury. We say large subject, because in this article, as in daily practice, the word neuritis is made to cover not merely inflammation of the nerves in question, but their atrophy and their degenerations. Indeed, the subject is rendered wider still by the facts that, on the one hand, peripheral neuritis usually results from some chemical poison which acts not on the peripheral nerves alone, but upon other parts of the nervous system; and, on the other hand, that affections of the nerves do not go on long without causing disorder in the parts to which they are supplied, so that disease of the peripheral nerves is apt to be complicated at both ends, if we may so say, by other morbid conditions. The whole article deserves the most careful study, for neuritis is the explanation of many chronic conditions which are all too apt to baffle the practitioner, and although no doubt at times these conditions may be incapable of cure, they not infrequently are the outcome of a chronic poisoning by some toxic material, the removal of which may pave the way to unexpected recovery.

Dr. Head contributes a very good article on Trigeminal Neuralgia, which he considers under the following heads: (1) Neuralgia quinti major; (2) neuralgia secondary to disease of the nerves of the head; and (3) neuralgia minor. He makes a strong point of the first of these being a definite disease of the nervous system, with a distinct course and character, standing quite apart from the other forms of pain in the fifth nerve.

The diseases of the other cranial nerves are treated on by Dr. Aldren Turner; then we have an article on Medical Ophthalmology, by Mr. Brudenell Carter; one on Diseases of the Vertebral Column, by Mr. Victor Horsley; and one on Affections of the Spinal Meninges, by Dr. Risien Russell.

Altogether this is an admirable volume, touching, no doubt, upon many subjects regarding which our knowledge is as yet far from being complete, but dealing with these subjects in such a manner as to be very explanatory, as well as setting out with considerable fulness the present state of our knowledge upon them.

